# Translating evidence in a priority setting partnership: knowledge gaps between healthcare providers and oesophageal cancer patients

**DOI:** 10.1007/s00520-022-07523-3

**Published:** 2023-01-21

**Authors:** Sarah McDonnell, Tara Breslin, Bianca Mascan, Nur Shuhada Shahruddin, Mawaheb Elnour, Michelle Fanning, Anthony Galvin, Jennifer Moore, Narayansamy Ravi, John V. Reynolds, Claire L. Donohoe

**Affiliations:** 1 School of Medicine, Trinity College Dublin, Dublin, Ireland; 2 Department of Clinical Nutrition and Dietetics, Trinity St James Cancer Institute, Dublin, Ireland; 3 Department of Surgery, National Centre for Oesophageal and Gastric Cancer, Trinity St James Cancer Institute, Dublin, Ireland

**Keywords:** Communication, Oesophageal cancer, Relationships, Education, Health information, Person-centred care

## Abstract

**Introduction:**

Despite the fact that health information is now more accessible than ever, knowledge gaps remain between patients and healthcare providers (HCPs). To date, the patients’ need for information following a diagnosis of oesophageal cancer has not been adequately met.

**Purpose:**

The purpose of this study was to identify why knowledge gaps exist between oesophageal cancer patients and HCPs and how to address them.

**Methods:**

Purposive sampling of a group of people living with and after oesophageal cancer who had participated in a priority-setting partnership where 45% of questions from patients had existing evidence-based answers. A 7-set question series was developed for use in a patient/HCP focus group in addition to 11 individual phone interviews with survivors of oesophageal cancer. Qualitative semistructured interviews were conducted to explore oesophageal cancer patients’ access to information. The data was analysed thematically, which involved coding all patient transcripts before identifying and reviewing key themes.

**Results:**

The three primary themes that emerged were as follows: *opportunity* (HCP team factors and relationship development), *ability* (patient factors) and *priority* (pacing of information delivery).

**Conclusion:**

Effective communication between patients and HCPs was identified as an integral component of the enhancement of patient knowledge. HCPs should continue to refine and improve methods of information delivery and encourage conversations regarding information preferences.

**Supplementary Information:**

The online version contains supplementary material available at 10.1007/s00520-022-07523-3.

## Introduction

Oesophageal cancer is a serious and life-altering malignant condition with a 5-year survival rate of less than 20% [1]. Person-centred care requires that people are supported to develop the knowledge, skills and confidence to understand and manage their health [2]. Ascertaining individual health information needs is a crucial part of determining what information people require in order to make decisions [3]. While health information has never been more accessible than it is today, knowledge gaps remain between patients and their healthcare providers (HCPs). Information needs and barriers to obtaining or seeking information have been identified in many previous studies in patients with gastrointestinal cancers [3–6].

Patients diagnosed with oesophageal cancer desire a significant amount of information regarding their illness [7], and healthcare professionals (HCP) tend to underestimate patients’ needs for information following a diagnosis of oesophageal cancer [8]. Researchers in our centre have previously conducted a priority-setting partnership for research into oesophageal cancer asking patients, their supporters and healthcare providers to identify key unanswered questions amenable to further research with the aim of generating the top priorities for oesophageal cancer research. Almost half (45%) of questions submitted by people living with and after oesophageal cancer were already answered by research. Therefore, this information would likely have been accessible to people living with or after oesophageal cancer if they had put these questions to their healthcare providers or accessed patient information resources. Thus, the purpose of this study was to examine a cohort with demonstrable gaps in their knowledge concerning their cancer, by exploring, in a qualitative fashion, their experience of information transfer and whether this met their needs. We sought to understand whether this deficit in knowledge was associated with a perceived difficulty in accessing information and/or an unmet health information needs.

From this exploration, it might be possible to ascertain what changes in practice might result in a reduction in this knowledge gap.

## Methods

Patients who participated in a priority-setting partnership, were recruited via social media, or opted into a research mailing list after treatment in a tertiary cancer centre were asked to provide contact details if they were amenable to participation in further research studies. Ethical approval for this study was obtained from the hospital research ethics committee and the data generated were stored anonymously in line with GDPR [9]. Participants were contacted by email or phone and provided with a brief description of the study and a written patient information leaflet was then emailed or posted to the participants. Participants were given a 7-day cooling-off period and then were re-contacted to obtain written informed consent. Participants were eligible for inclusion if they had previously been diagnosed with locally advanced or metastatic cancer of the oesophagus and underwent treatment for the condition. Exclusion criteria included participants treated with endoscopic therapy only or supportive treatments only.

Recognising that all participants reported unanswered questions about oesophageal cancer and almost half of these questions had evidence-based answers (and therefore did not reflect evidence uncertainty), three hypotheses were generated by the research group to propose reasons why there was a deficit in knowledge in patients.

- *Resources available but unable to utilise* (shock and fatigue, social/family support, volume of information, geographical location, educational background, patient mindset, timing of information delivery)
- *Communication barriers between HCP and patients and among HCPs* (i.e. continuity of care, accessibility, use of open communication)
- *Efficacy of the patient-HCP relationship* (patient willingness to discuss diagnosis/treatment related questions or concerns with HCP, volume of information, continuity of care)

We tested these hypotheses by developing a 7-set question series in consultation with a mixed patient/HCP focus group prior to individual patient interviews (Supplementary Table 1) to ensure that wider patient perceptions were incorporated into the questions asked during individual interviews. Three survivors of oesophageal cancer and other allied health professionals (1 surgeon, 1 dietician, 1 nurse specialist, 1 researcher) participated in a qualitative focus group conducted via Zoom. The focus group followed a semi-structured guide that focused on the participants’ experiences throughout their diagnosis, treatment and survivorship and HCP experiences of treating patients with oesophageal cancer. Patients were asked to reflect on their own experience learning about their condition and describe how they sought information about their condition at that time. They were asked to consider factors that helped or hindered them in meeting their information needs (i.e. visual aids, geographical location, support, technological aptitude). The question set was then tested on the patient participants. Following the focus group, the set of 7 questions was refined to broaden the scope of the interview. Two questions were combined as they achieved the same response, and one new question was created (Supplementary Table 1). The focus group reported that the questions adequately addressed possible causes of knowledge deficits and that the introduction describing the purpose of the study and questions was clearly understood.

Qualitative semistructured interviews were then conducted by the research team. Purposive sampling generated participants who were survivors of oesophageal cancer, had participated in the priority-setting partnership, and had previously agreed to be contacted for future research. Participants were selected at random and contacted by email. Following informed consent, 11 structured phone interviews were conducted by 5 members of the research team, who interviewed 2–3 patients each, using the same set of questions (Table 1). An initial “ice breaker” exercise was conducted in which patients were asked to rate their preference for having access to information using 5 health-related questions and a modified information preference scale (IPS) [10] (Table 2). The set of 7 questions developed from the hypotheses was utilised to prompt the researcher to discuss key themes. Patients were asked to reflect on their own experience learning about their condition and on how other peoples’ experiences might differ from their own (if they did not personally have difficulty gaining information or if they did have difficulty, why that might have been).

**Table 1:** Patient demographics

Patient number	Age	Gender	Location (urban/rural)	Highest level of education	Year of cancer diagnosis
1	68	Male	Semi-rural	Higher	2018
2	72	Male	Rural	Secondary	2017
3	70	Female	Urban	Secondary	2009
4	70	Female	Rural	Higher	2017
5	44	Male	Rural	Higher	2016
6	62	Male	Rural	Secondary	2011
7	85	Male	Urban	Higher	2015
8	49	Male	Urban	Secondary	2018
9	67	Male	Urban	Secondary	2010
10	65	Male	Sub-urban	Higher	2016
11	65	Male	Urban	Secondary	2016

**Table 2:**
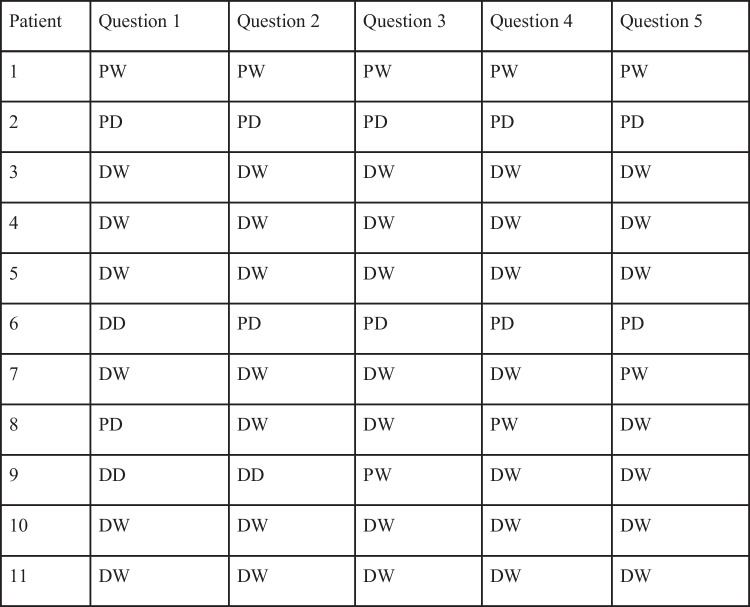
Modified IPS measure for information preference

Interviews lasted 20–60 min (median length = 31 min), were conducted in English and were scheduled according to the participant’s preference. Interviews were audio-recorded using a recording app for Android/iPhone and transcribed using the Trint App transcription feature with the consent of the patient. Indirect or direct identifiers were removed from the transcript.

A thematic analysis was conducted according to the approach described by Maguire and Delahunt [11]. The data determined the themes that were generated. The data were analysed by two different members of the research team, both of whom had not conducted the initial interview. The transcripts were first reviewed to establish familiarisation with the data, and then a second time for the application of specific codes to the text. For example, the patient statement “I was very satisfied” received the code *patient experience*, in order to assign context. Several other codes were generated including *overall experience*, *relationship with the healthcare team*, *delivery of information*, *patient attitude and access to resources.* A third review assessing for unlabelled themes was conducted of all transcripts by the senior author (CLD).

**Fig. 1:**
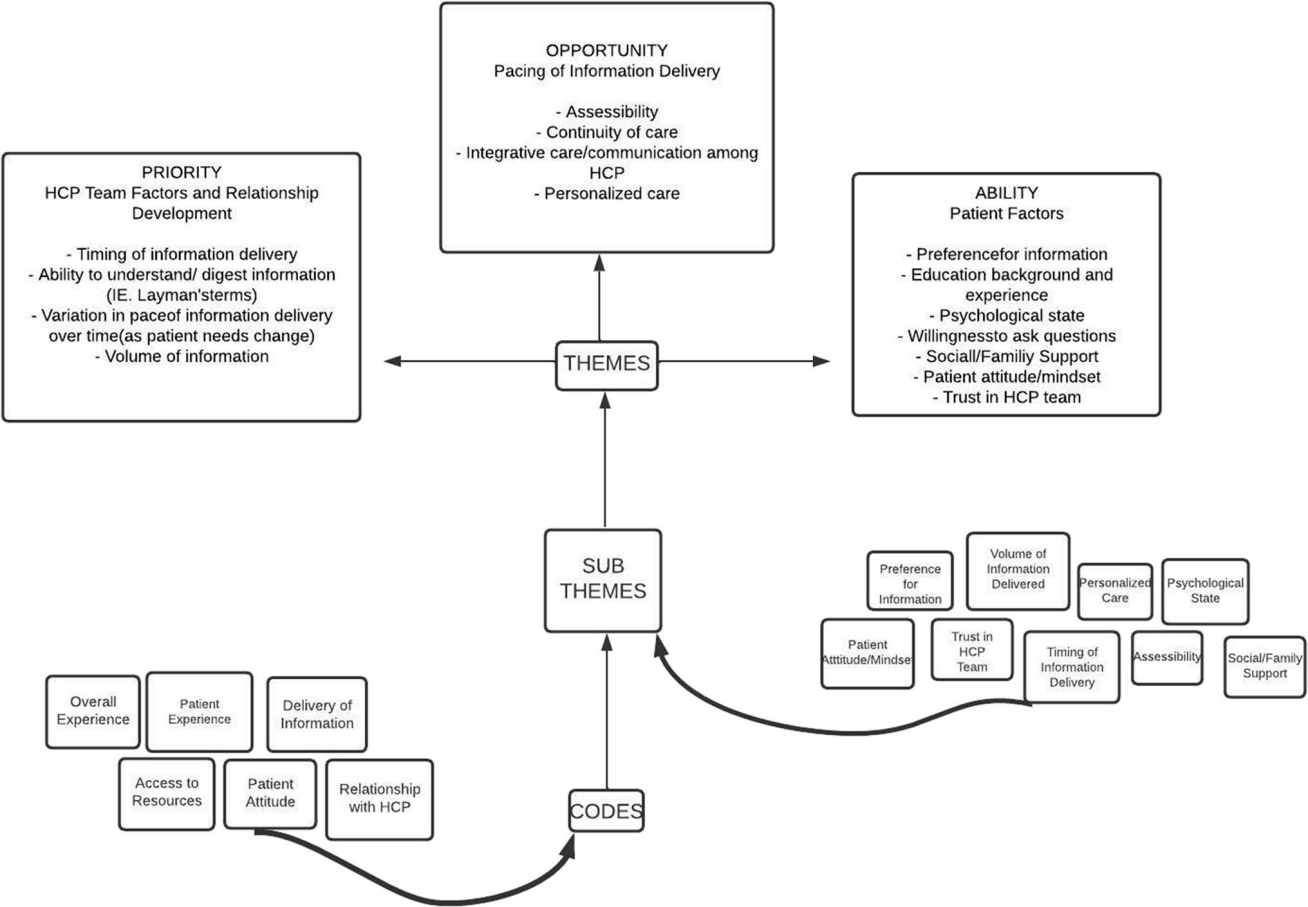
Development of primary themes from secondary subthemes and codes

Once all transcripts were coded, subthemes were identified. For example, when examining the code of *patient experience*, it was identified that patients expressed a range of information preferences, which ultimately influenced their experience as a patient. This finding was assigned to the subtheme *preference for information.* Patient statements were taken directly from interview transcripts to support each subtheme. Once all subthemes were identified and patient quotes selected, they were grouped together into larger primary themes based on compatibility (Fig. 1). Three primary themes were developed to represent the secondary subthemes in consolidation with the original research hypotheses (Fig. 1). All primary themes, related subthemes and supporting interview statements were organized into a table (Table 3). The three primary themes: *opportunity* (HCP team factors and relationship development), *ability* (patient factors) and *priority* (pacing of information delivery) were examined to gain an understanding of patients’ experiences. Additionally, specific factors reported by patients in helping meet their information requirements were assessed. A table was generated to demonstrate instances of good practice within each identified theme (Table 4).

**Table 3:** Primary themes and associated patient statements

Ability → patient-related factors Preference for information “I never, ever wanted to know one thing about the operation. I just didn't want to know the gory details. I didn't look it up on Google and I didn't want to talk about it.” Patient 2 “I’m not a drama queen…tell me the truth because then you can deal with it.” Patient 8 “I preferred the approach the surgeon gave me when he said it's inoperable and incurable, because it was direct and honest. I don't like people beating around the bush.” Patient 9 “When I was initially diagnosed, I wouldn't have been able to handle the information that was considered painful but useful, but now that has changed, I would actually prefer the physician and the health care team have to have the direct approach.” Patient 9 “I am a person who always wants to know all the information.” Patient 4	Opportunity → HCP team–related factors and relationship development Continuity of care “The follow-up visits with the team were essential for both my mental and physical health.” Patient 6 “The physios are in the hospital and they were around to walk me around the wards but they never actually told me what I was supposed to do or continue to do. I didn't get any sort of information, I was just told by the doctors and nurses, and the dieticians to walk and eat small but plenty and all of this.” Patient 9 “I was going to X private hospital, and my surgeon in Y hospital did the transfer of information due to COVID from the public hospital.” “They were very good, anytime I went to Y hospital they sent information over to my other oncologist. They had the results of any treatments or scans, and while I was in Y hospital, she even contacted me on the phone about things…whatever was done in one hospital was transported to the other hospital.” Patient 4	Priority → pacing of information delivery Patient ability to understand and digest information “I think in hindsight the information was there, but I didn't fully understand it.” Patient 1 “It hasn't been distilled down, some of it is yet to be put into layman's language. So, I was trying to use my limited science knowledge to interpret some of it.” Patient 1 “If there was one barrier (to accessing resources) it was my ability to interpret all the information. The biggest barrier would have been my ability to interpret and absorb everything.” Patient 1 “I think knowing the information really helps, so when you know what’s important and you need, you're more likely to adhere to the regiment.” Patient 4
Education background & experience “As an allied healthcare professional, I have quite a bit of information. I suppose the fact that I knew a bit, what I was taking the day before I went along because I understood what they were telling me.” “I would probably have asked fewer questions if I worked in a different field.” Patient 4 “I would say the fact that I'd been through major health issues before may have geared me better to managing the flow of information to and from the various doctors and specialists I was dealing with.” Patient 10	Accessibility “Anything that I ever had to talk to her about, she was there. If she didn't answer, the phone would ring later. She'd always get back, you know, which was very reassuring.” Patient 2 “I can talk to my liaison (by text), she does a fine job on her busy day texting me back quickly…and that’s all I want….I could approach any of them no problem at all.” Patient 8 “She (liaison nurse) made everything more bearable, enabled me to feel at ease.” “I couldn’t praise her (liaison nurse) enough.” Patient 3	Volume of information “I felt it was such a huge dump of information, I was trying to process it all, you know, and on top of that, just trying to deal with the situation that you're emotionally trying to deal with, the fact that you have cancer…But, you're trying to process all at the same time.” Patient 1 “He didn't give me a huge amount of information. Just enough, enough to keep me sorted, you know.” Patient 10
Psychological state “But the initial diagnosis, it was a bombshell, I was walking around beside myself.” Patient 9 “Everybody’s different and everybody suffers in different ways and is mentally affected in different ways.” Patient 8		Timing of information delivery “His approach was very good, was clear, it was concise, and the information he gave us was timely. He didn't give us all the bad news at the start.” Patient 10
Geographical location (rural vs urban and proximity to home) “I didn’t go down the road of support groups but that was my own personal choice. I mean I don’t drive. That was kind of a factor (but I didn’t feel I was losing out because of that.) It was just my own personal choice.” Patient 3 “I only found out about the services and accommodation around 18 months after my surgery, I had none of that for the duration of my treatment.” “I didn't know any of that existed especially for people in rural parts like myself.” Patient 9	Personalized care Doctor “X” was accommodating of Patient’s own goals- “he come back into me the next day and he said, so you want to go back to work…we better start working on that and they gave me all the advice”	
Social/family support “X was there, and she wrote down all the information.” Patient 6 “My partner is a retired allied healthcare professional and to me that was the key because I didn’t understand the medical terms…I'd never been sick…” “My wife does more of the reading for me and then explains it to me.” Patient 8 “I thought I was going to have a nervous breakdown; my wife was the person I turned to.” “I turned to my friends and wife.” Patient 9	Independent patient research “A lot of people use Doctor Google and now they're beginning to see that it has benefits… you know if it's used in the right way.” Patient 1 “The doctor said, you need to be careful with this kind of stuff because you might be getting stuff that isn't really medically sound.” Patient 10	
Patient attitude and mindset “I had no problem taking any advice that I got there…and I kind of had to reinvent myself in a way in the eating habits and whatever…it was very tough in the beginning” Patient 7 “For me the big thing is you’ve got to be disciplined with yourself…” Patient 7 “Positivity will get you through a lot of it too…” Patient 8 “Some people are the kind of people that would find faults even if things were perfect…” Patient 3		
Trust in HCP “You’re only as good as what the medics tell you, if you listen to them.” Patient 7 “I'm happy when I have the oncologist or surgeon tells me what's happening and basically told me this is what's wrong and this is what needs to be done.” Patient 8		
Willingness to seek clarification on unanswered questions or concerns “It wasn't properly made clear that if you didn't understand it, maybe ask your doctor.” “I think in hindsight the information was in there, but I didn't fully understand it.” Patient 1 “Nobody told me to come down off them (morphine patch) gradually. I suddenly felt really depressed and I was crying…I just didn’t know. It was just ignorance on my part.” Patient 3 “I know that people might not be as vocal and might not get as much from it, and it's a more difficult experience for them.” Patient 8

**Table 4:** Examples of good practice within each identified theme

Ability → patient factors	
Verbal information delivered by trusted member of HCP best source of information	“He (professor) kindly went over the diagnosis with me again, you know, very caring and effective.” Patient 1 “For me, it was mainly verbal information.” Patient 2
Supplementation of verbal and written resources with visual modes such as diagrams and videos were effective and informative interactive strategies	“They had a CD; I actually have it here. The patient is shown it, and it's the oesophageal cancer treatment. I found that very good.” Patient 9 “I particularly found the video really good because it gave me a visual image.” Patient 10
Benefit of shared patient experience to information access	“It (support group) was very good to go there to see other people and see how they responded to different things.” Patient 2 “Information sessions sharing talks on the latest state of knowledge on oesophageal cancer, what the treatments are, what the possibilities are. People who are at different stages of the illness are getting together for coffee mornings and just sharing their experiences.” Patient 10
The impact of positive attitude/mindset on patient experience	“I went within minutes of having a poor, desperate diagnosis to say look, I'm positive. What can I do to make sure that my journey is going to be the best possible one going forward?” Patient 1 “Positivity will get you through a lot of it too I have to say. I believe anyway.” Patient 8
Opportunity → HCP team factors and relationship development	
Continuity of care provided by MDT post-treatment	“It's been continuous, I’ve had regular scans, and contact and when I had the re-occurrence, I had to go to surgery the second time round and I found the multidisciplinary team very helpful. The whole team was brilliant.” Patient 4 “The follow-up visits with the team were essential for me, for both my mental and physical health.” Patient 6
Consistent access to information and availability of MDT to address patient questions/concerns	“If you had anything to worry about, you dropped a phone call.” Patient 1 “I had a phone number available 24 h. The professor and his secretary were great. Very, very helpful.” Patient 2 “If she didn't answer, the phone would ring later. She'd always get back, you know, which was very reassuring.” Patient 2
Patient self-reflection on the relationship shared with the MDT	“I couldn't say one bad word about them. I'm talking from the head man down to the person making the beds. I couldn't ask for better.” Patient 2 “Everybody was just second to none, really. I couldn't say no fault whatsoever.” Patient 3 “I can't say enough. I could be here telling you all day how wonderful you all are. And that is just a fact.” Patient 7 “There was no stone unturned.” Patient 2
Priority → pacing of information delivery	
HCP delivery of information tailored to individual patient needs	“The timing of the information was apt because I’m not so sure we’d have been able to handle that information just after the surgery.” Patient 10 “He broke it up into bite sized milestones.” Patient 10
Quality and quantity of the information accessible to patients	“The resources that were made available by the team were massive. And you kind of felt that. Nothing was left off the table in terms of your treatment, you know.” Patient 10 “I had a recent scare and he would call me or email me on a Sunday, on his day off, just to make sure that I was okay.” Patient 5
Personalization of care by the MDT to suit patient needs	“He (professor) came back in to see me the next day and he said, so you want to go back to work…we better start working on that.” Patient 7

## Results

Thirty-three patients from the PSP confirmed their willingness to be contacted again in the future. Twelve patients were randomly selected and contacted to participate in the study. Eleven patients agreed to participate (mean age = 65 years old, 9 male). Five resided in an urban location, 4 rural, 1 semi-rural and 1 sub-urban. Over half of the participants (54.5%) completed their secondary education, while 45.5% obtained higher education.

The IPS measure for information preference (Table 2) concluded that the majority of the patients interviewed would “definitely want to know” information if it was made accessible to them. A total of 54.5% of the patients answered “definitely want to know” when asked if they would want to know how long they were expected to live. Information preferences according to the type of information were generally consistent for each participant (i.e. health-related preferences were the same as general information preferences) (Table 2).

Having found that, contrary to the study hypothesis, having unanswered questions does not correspond with unmet information needs, we examined the data to ascertain why patients in this setting, with knowledge deficits, remained satisfied with the information that they sought and received during treatment and survivorship. Three primary themes were developed from the thematic analysis.

### Ability: patient-related factors

The data identified that most patients felt that they received adequate information regarding their condition and that there were no barriers to accessing information. Patients demonstrated a spectrum of information preferences. One patient described not wanting to know any information about the procedure: “I never, ever wanted to know one thing about the operation. I just did not want to know the gory details. I didn't look it up on Google and I didn't want to talk about it” (Patient 2). In contrast, another patient chose to conduct independent research: “I am a person who always wants to know all the information” (Patient 4). The results from the IPS corresponded with these findings.

Patients’ educational background and previous experience within the healthcare system may impact their ability to understand and interpret the information communicated to them. One of the patients stated that they believed that their prior history of health issues better prepared them for the delivery of information: “I would say the fact that I'd been through major health issues before maybe geared me better to managing the flow of information to and from the various doctors and specialists I was dealing with”(Patient 10). Other patients also highlighted that their geographical location and proximity to services like support groups impacted access to information: “I didn't know any of that existed especially for people in rural parts like myself” (Patient 9).

Family and external support was discussed by multiple patients: “X” was there, and she wrote down all the information” (Patient 6) and “my partner is a retired allied health practitioner and to me that was the key because I didn’t understand the medical terms…I'd never been sick…” (Patient 8). There was also a range of patient attitudes demonstrated between interviews. Patients who reported no unanswered questions also demonstrated traits of resilience and a positive mindset. One patient stated that “positivity will get you through a lot of it…” with reference to his cancer journey (Patient 8). The patients who reported greater trust in their HCP and MDT also reported that most of their questions were answered. Similarly, those that described a trusting relationship with their HCP believed that they had access to all the information they needed. A patient remarked on his satisfaction: “the oncologist or surgeon told me what was happening and basically told me this is what's wrong and this is what needs to be done” (Patient 8).

Patient willingness to seek clarification was identified as a determinant of information access. A patient who did not seek clarification stated he was unaware that he could ask questions. “It wasn't properly made clear that if you didn't understand it, maybe ask your doctor” (Patient 1). Another patient acknowledged how individual differences could also account for this hesitation. “I know that people might not be as vocal and might not get as much from it, and it's a difficult experience for them” (Patient 8).

### Opportunity: Hcp team–related factors and relationship development

Patients reported fewer unanswered questions when there was continuity of care, personalised care and when an HCP was accessible (i.e. phone, text, e-mail). One patient reported that continuity of care post-treatment was critical to their recovery and access to information. “The follow-up visits with the team were essential for both my mental and physical health” (Patient 6).

Most of the patients reported sufficient continuity of care. One patient described: “anytime I went to Y hospital they sent information over to my other oncologist. I think the X private hospital has the file on computers now anyway, it's an easier method to make sure that your information goes forward and back easily”. In contrast, failed continuity of care caused another patient to feel unsure of the next steps. “The physios were in the hospital and they were around to walk me around the wards, but they never actually told me what I was supposed to do or continue to do. I didn't get any sort of information” (Patient 9). In some cases, offering personalised care led to fewer unanswered questions and a more positive experience overall. In one interview, the patient reported that the doctor was accommodating to the patient’s personal goal to return to work: “he came back to me the next day and he said, so you want to go back to work…we better start working on that and they gave me all the advice” (Patient 7).

### Priority: pacing of information delivery

There were fewer unanswered questions when the information provided to the patient was in terms that the patient could understand. Patients reported a greater sense of understanding when the information was tailored to individual preferences and properly paced with changing information requirements over time. The volume of information and timing of delivery played a role in whether or not patients were able to adequately interpret the information. “Information overload” impeded some patients from getting answers to their questions.

When discussing barriers to accessing available resources, one patient stated that “if there was one barrier (to accessing available resources) it was my ability to interpret all the information…my ability to interpret and absorb everything” (Patient 1). It was mentioned by another patient that understanding the information that they were provided ensured their compliance with treatment. “I think knowing the information really helps. You're more likely to adhere to the regimen because you understand how important it is” (Patient 4).

Patients were more satisfied when the HCP team ensured that the delivery of information was appropriate in volume and timing. One patient described their HCP approach as “very good, it was clear, it was concise, and the information he gave us was timely. He didn't give us all the bad news at the start”. This patient also reflected positively on his experience stating that the HCP team provided “ just enough (information) to keep me sorted” (Patient 10). In contrast, another patient described: “I felt it was a huge dump of information, I was trying to process it all, you know, and on top of that, trying to deal with the situation emotionally, the fact that you have cancer… you're trying to process it all at the same time” (Patient 1).

Multiple patients reported that face-to-face communication was the most effective method of information delivery. Supplementation of verbal communication with visual and written resources (i.e. videos, pamphlets) enhanced patient access to information and bridged gaps in understanding. Social support groups and awareness of these additional resources improved access to information by facilitating the sharing of experiences and information between patients.

Several patients reported that HCPs went above and beyond to ensure that they had access to any information/resources that they needed. Communication among HCPs provided smooth transitions of care and minimized the number of unanswered patient questions. HCP accessibility, specifically a dedicated team member available to patients, provided a constant source of information and an open line of communication between patients and their HCP.

Fewer questions were left unanswered when the volume of information and pacing of delivery was timed to suit the individual patient’s needs. HCP consideration for patient information preferences strengthened trust and improved personalization of care.

## Discussion

Information needs and barriers to accessing information have been reported in many studies of patients living with and after cancer diagnoses [12, 13]. Despite sampling from a cohort of patients in whom there was a demonstrable self-reported deficit in knowledge about their cancer, the majority of patients who participated in this study reported that they received adequate information regarding their condition and experienced no limitations to accessing information. Patients were most satisfied when the information that they received was delivered by a trusted HCP and tailored to their individual needs. Although these people living with and after an oesophageal cancer diagnosis had unanswered questions, many of which could have been answered by their healthcare providers or using patient information resources, participants did not report that their information needs were unmet. All reported a range of remaining questions about oesophageal cancer but this did not correspond with reporting dissatisfaction with the amount of information conveyed.

This study demonstrates that there is a difference between being informed and feeling informed. Rather than finding that patients with demonstrable knowledge deficits feel ill-informed, participants were satisfied with their level of knowledge and the information that they received. Information transfer is not merely about conveying factual information and therefore best tested by a recall of facts. Patient-reported outcomes such as patient-perceived trust and satisfaction with information transfer are arguably more important measures of successful communication. The measure of successful communication in this setting is ensuring that people have enough information to enable them to participate to their desired level in their healthcare. This study provides evidence to support the conception of person-centred care as enabling, personalised and coordinated [2].

Shared decision-making is described as the crux of patient-centred care [14]. It requires that patients have an adequate understanding of their condition to weigh treatment choices and arrive at a treatment plan that meets their individual goals of care [15]. Neither the operational definition for shared decision-making [16] nor the tools for its implementation [17] are comprehensively described. Implementation of shared decision-making in real-world clinical practice is hampered by the lack of these tools, and even when directly studied, patient participation in the process is low [18]. How much information is required during this process depends on the individual patient’s information preferences [12, 18].

Patients can be represented as existing on two spectra of information preferences — monitoring and blunting. “High monitors” are information seekers, utilising active information-seeking behaviour in order to access information compared to “low monitors” who are more passive. Distractors or “high blunters” avoid stressful or negative information with non-distractors at the opposite end of the spectrum [19]. Satisfaction with information needs being met is reported as reduced amongst those who are a combination of high monitors and low blunters [19].

Participants in this study reported a range of information preferences and seeking behaviour, representing different self-reported monitoring and blunting behaviours. All were treated by the same treatment team and yet reported satisfaction with information delivery regardless of their preferences and tailoring of information to meet their individual needs and desires to participate in their healthcare.

Due to personal preferences for information, some patients elected to receive only a select volume of information while others sought out as much information as possible — variation which is typically seen in clinical practice [20]. Yet despite different health information–seeking behaviours, this cohort was satisfied with their understanding of their condition and the information that they possessed. Therefore, this study shows that the aim of patient counselling is not to just provide factual information so that there remain no unanswered questions, but instead to provide adequate information such that patients are satisfied that they have met their information needs and can readily access further information, as required. This study enables us to analyse factors reported by patients as enabling them to satisfy their individual information needs, even in the context of a demonstrable knowledge gap between patients and their HCPs. Thematic analysis of patient transcripts revealed three factors that influenced the experience of information transfer: individual patient factors that influence their ability to access and acquire information: pacing and volume of information delivery and opportunities to develop a trusting relationship with the healthcare provider team.

One key theme was the development of a longitudinal relationship between the patient and their team of HCPs. All participants remained under review by their treatment team at the time of the study. Without prompting, all participants chose to discuss their experiences of information transfer at the time of diagnosis and during treatment rather than discussing experiences of knowledge requirements when living with or after the disease. One of the themes that emerged was having an ability to make contact with the healthcare team and perhaps this ongoing relationship influenced the perception of met information needs. Previous studies have reported that information-seeking behaviour does differ according to a phase of treatment and that patients are more likely to report passive information-seeking behaviour after treatment ends [21, 22]. Satisfaction with information delivery may similarly increase as the interval from the time of diagnosis increases. Coordination and continuity were enablers of the relationship.

Patients reported having a greater sense of support and continuity of care when an HCP or associated team member was available and easily accessible (i.e. by text/ voicemail/ phone call). Most patients described the relationship with their HCP team as positive. Some patients also highlighted the relationship with the cancer nurse specialist specifically, who was not only instrumental in ensuring that patients felt heard by addressing questions/concerns promptly but also communicated effectively within the HCP team. This study adds to the extant small body of literature reporting the beneficial role of the clinical nurse specialist in information delivery and education [23]. In addition, this study reports that the clinical nurse specialist role is complementary to and supplements information provided by other team members — particularly in enabling patients in accessing the information they sought. Continuity of care, access to resources and patient experience were improved with efficient communication among HCPs both in the hospital and at the community level. Trust in healthcare providers to provide required information was prevalent in this study and reported elsewhere [24]. Levels of trust correlate with perceived good communication abilities in doctors in many studies [25].

Tailoring the amount and timing of the information given to a patient can positively influence the patient’s perceptions of information transfer. Attempts at ameliorating a knowledge gap might be unintentionally exacerbated by providing too much information at one time. The HCP must carefully consider this so as not to overwhelm the patient. D’ haese et al. found that a step-wise delivery format reduces patient anxiety [26] aligned with control and hope as a factor which can influence satisfaction with information access. Patients in this study reported that factors other than previously identified standard demographics [27] influenced their satisfaction with communication. Participants identified personal traits of resilience and a positive mindset as important influences on their satisfaction with information transfer. Dispositional optimism has been shown to be associated with improved self-reported health-related quality of life, reduced symptom severity and psychological distress. It may also be associated with an enhanced perception of HCP communication and relationships [28, 29]. Dispositional optimism has been associated with risk perception and associated information-seeking behaviour — optimists may perceive less risk and are therefore more likely to actively seek genetic testing results [30]. Further studies from other centres may prove useful to corroborate this finding — that patient’s generalised tendency to expect good outcomes in important life domains may also influence their information preferences and perceptions of information transfer.

Experiences reported in this study emphasised the quality of the relationship that develops between HCPs and patients as an influence on information-seeking behaviour, especially from other sources. Information carrier factors in the CMIS are categorised by characteristics and utility. Johnson et al. reported that the characteristics of information carriers preferred included information from other people or channels that reproduce person-to-person interactions. Participants in this study congruently emphasised that face-to-face interaction was paramount to a strong relationship with their HCP and improved access to information — a factor which may have been of increased salience during the time when this study was conducted — after over 1 year of lockdowns and decreased access to in-person contact with healthcare professionals [31, 32]. Characteristics such as trustworthiness and credibility were important both in personal interactions and other media in Johnsons’ model. In this study, the relationship with the HCP team also influenced the choice of other knowledge sources accessed, as participants reported information sources provided by or sign-posted by HCPs to be more valuable than those found through independent searches. This was attributed by participants to the utility of information from HCPs as it was perceived to be tailored to and therefore relevant to the individual participant. The conception of the information carrier’s qualities as objective or independent of their relationship with the patient in CMIS could overlook a modifiable factor in clinical practice. Patients who perceived a positive inter-personal relationship with the team and consequently perceived their care as personalised [24, 27–29, 33]. Previous studies have indicated that information-seeking behaviour can be partly determined by the patient’s perception of their healthcare provider as an information giver and described different approaches to information-seeking when the patient is uncertain about the authority of their physician as an information giver [33]. Thus, consideration for the quality of the relationship that develops between patient and their HCPs is fundamental to the perception of person-centredness.

There are several limitations and potential sources of bias in this study. Patients self-selected to participate in further research and all were satisfied with the care they received. All the participants felt that they received sufficient information based on their personal preferences, and as such, this study does not address the experiences of patients who do not meet these criteria. There may also be cultural issues associated with how Irish people view healthcare professionals, as there is a strong theme of trusting the doctor or team, and by extension, there may be a hesitancy to challenge or ask questions. This study cannot address the influence of its cultural context on the findings reported and this may limit the generalisability of the findings, not being an ethnographic study by design.

Interviews were conducted by medical students and as a result, patients may have felt it important to speak positively about their care to those in training. This study was also conducted during the early phases of the COVID-19 pandemic when public responses to healthcare professionals may have been more positive than usual [34]. Although there was a range of ages and education represented, there was limited diversity within the cohort and doctor communication styles are known to vary according to ethnicity and socioeconomic status [27, 31, 32].

Perhaps surprisingly, no themes specific to oesophageal cancer or poorer prognosis cancers in general emerged from the thematic analysis. Patients did not allude to the relatively poor prognosis of this particular cancer type nor discuss how information-seeking behaviour or needs might differ with this type of cancer compared to other cancer types or indeed other healthcare conditions. Patients were specifically prompted to focus on their personal experiences and opinions about information acquisition at the beginning of the interview and this may account for the lack of reflection on the specific context of oesophageal cancer. Participants did not report frequently accessing online or other patient information materials in this study, other than those provided or sign-posted by the clinical team or dedicated support groups. This may be related to the disease being an infrequently diagnosed cancer, and perhaps the relatively poor prognosis and an example of distorting/blunting [19] protective information behaviour.

The HCP must come to understand individual patient needs and encourage dialogue regarding information preferences. Understanding the individual goals and values of patients, as well as the external factors that influence how patients understand their health, may allow HCPs to make evidence-based recommendations individualised to the specific needs of a patient.

## Conclusion

Health information is now more accessible than ever; however, knowledge gaps remain between patients and healthcare providers (HCPs). Despite this, patients reported that their information needs were met. The findings of this study confirm that patients demonstrate a spectrum of preferences in terms of the disclosure of information. Health literacy and previous experiences may impact patients’ ability to understand and interpret information. Family and social support were beneficial in relaying information to the patient, prompting additional research and sourcing questions. Patient resilience can either positively or negatively affect the ability to absorb and interpret information, as patients reporting their information needs were met, self-reported traits of resilience and a positive mindset. The importance of tailoring information provided to individual patient preferences and proper selection by HCP of when to deliver information also proved beneficial, as this led to fewer unanswered questions.

### Practical value

The findings of this study have several implications. People living with and after cancer diagnoses live with unanswered questions about their disease but this does not necessarily correspond with a perception of ongoing information needs or a deficit in knowledge. Factors that are reported by patients to help meet their information needs include the ability of individual patients to seek and understand information (ability), a relationship with healthcare providers that promotes individualised knowledge sharing (opportunity) and receiving information that is personalised and relevant to the person at the time (pacing). These themes are congruent with conceptual definitions of person-centred care as enabling, coordinated and personalised [2].

Future research should focus on understanding the experiences of patients who report their information requirements are not met.

## Supplementary Information

Below is the link to the electronic supplementary material.

Supplementary file1 (DOCX 15 KB)

## Data Availability

The authors confirm that the data supporting the findings of this study are available within the article and its supplementary materials.
