# Supplementary material for: Translating evidence in a priority setting partnership: knowledge gaps between healthcare providers and oesophageal cancer patients
Source: Support Care Cancer. 2023 Jan 21;31(2):126. doi: 10.1007/s00520-022-07523-3 (PMC9860237; doi:10.1007/s00520-022-07523-3)
Supplement: Supplementary file 1 — Supplementary file1 (DOCX 15 KB) [file 520_2022_7523_MOESM1_ESM.docx]

### **SUPPLEMENTARY INFORMATION (SI)**

**Table I: Outline of Interview Structure**

| **Who we are and reason for interview**  **General demographic questions:** Age, Gender, Location (urban/rural), Highest level of education (primary, secondary, third level, higher degree), Year of cancer diagnosis  **Modified IPS measure for Information Preference**  An exercise to rate your preference for information. This exercise looks to see if you are the kind of person that likes to know a lot of details and all of the information regardless of its emotional affect or if you are someone who only wants some of the details. ***I will ask you 5 questions and if possible, please respond with “Definitely Don’t Want to Know, Probably Don’t Want to Know, Definitely Want to know, or Probably Want to Know.”***  1. As part of a semi-annual medical check-up, your doctor asks you a series of questions. The answers to these questions can be used to estimate your life expectancy (the age you are predicted to live to). Do you want to know how long you can expect to live?  2. You provide some genetic material to a testing service to learn more about your ancestors. You are then told that the same test can, at no additional cost, tell you whether you have an elevated risk of developing Alzheimer’s. Do you want to know whether you have a high risk of developing Alzheimer’s?  3. At your annual check-up, you are given the option to see the results of a diagnostic test, which can identify, among other things, the extent to which your body has suffered long-term effects from stress. Do you want to know how much lasting damage your body has suffered from stress?  4. Some people seek out information even when it might be painful. Others avoid getting information that they suspect might be painful, even if it could be useful. How would you describe yourself?  5. If people know bad things about my life that I don’t know, I will prefer not to be told. |
| --- |

| **Questions Developed from Hypotheses**  1. What method of communication (booklets, online resources, etc.) do you think is most effective in delivering information to patients? Were you able to easily understand the information provided?  2. How would you describe the relationship that you shared with the HCP team during your treatment? Did you feel like this relationship continued even after your treatment? With whom did you feel most comfortable discussing your diagnosis/treatment/etc.?  3. Do you feel there is anything else your HCP could have done to make you feel more comfortable sharing your questions and concerns?  4. What about your journey did you find to be the greatest barrier to accessing available resources? Do you think there is anything more that HCP can do to make sure you are still able to access these resources?  5. Did your questions arise during your treatment, or months/years later? Could the answers to these questions have impacted your journey in any way?  6. Could you reflect on why other people's experience might differ from yours? For example, if you didn’t have difficulties personally, why do you think that was? (e.g. I trusted what my doctor told me). Also, if someone did have difficulty (e.g. feeling comfortable talking to their HCP) why might that be (even if they didn't experience this themselves).  7. Can you think of any specific ways that information could be shared more effectively with patients? (I.e. making sure all patients had contact details for a patient support organisation etc.) |
| --- |
